# Electromyographic Activity and Applied Load During High Intensity Elastic Resistance and Nautilus Machine Exercises

**DOI:** 10.2478/v10078-011-0067-0

**Published:** 2011-12-25

**Authors:** Saied J Aboodarda, Mohamad A.H. Shariff, Ahmad Munir Che Muhamed, Fatimah Ibrahim, Ashril Yusof

**Affiliations:** 1 Sports Center, University of Malaya, Malaysia; 2 Faculty of Medicine, University of Malaya, Malaysia; 3 Advanced Medical and Dental Institute, University Sains Malaysia, Malaysia; 4 Dept of Biomedical Engineering, Faculty of Engineering, University of Malaya, Malaysia

**Keywords:** electromyogram, elastic tubing, variable resistance training, multiple repetitions maximum

## Abstract

This study was designed to quantify and compare Electromyographic activity (EMG) and applied load in quadriceps muscle within performing high intensity knee extension exercises by Elastic Resistance (ER) and Nautilus Machine (NM). Sixteen male and female subjects (22.4 ± 4.7 yrs) completed 8 RM seated knee extension by NM, elastic tubing with original length (E0) and elastic tubing with 30% decrement of original length (E30). The mean value of EMG and external force were calculated and synchronized across various segments of motion for the three modes of training. The results demonstrated that in the early concentric and late eccentric segments of contraction, NM elicited significantly higher muscle activation than both E30 and E0 (p < 0.05). However, in the mid-concentric and mid-eccentric as well as late concentric and early eccentric segments no significant differences were observed between NM and E30. These findings supported the approach that developing external recoil of force in ER device by reducing 30% of initial length of elastic material can offer similar neuromuscular activation compared with NM. On this basis, E30 can be suggested as an affordable and non-gym based exercise device which has the capacity to provide an appropriate high resistance stimulus to meet the training requirement of athletes.

## Introduction

Resistance exercises have become an inevitable part of training schedule that is considerably recommended to develop musculoskeletal health and fitness (ACSM, 2002). The three modalities of apparatuses that are typically used to perform resistance training are free weights, pulley machines and isokinetic dynamometers. Despite extensive application, the use of free weights and pulley machines has been widely controversial; because, these training devices provide constant external force rather than constant muscular tension throughout the range of motion [(ROM), (Fleck & Kraemer, 2004; Wallace et al., 2006)]. In theory, the provided external resistance must be accommodative to result in maximal muscle stimulation across the whole ROM (Elliott et al., 1989; Manning et al., 1990). However, the resistance training apparatus which can meet the above criterion, the isokinetic dynamometer, is very costly and often used in rehabilitational setting.

Based on these disadvantages, the attention of athletes and coaches has been directed toward what is now known with the term Variable Resistance Training (VRT). Manning et al. (1990) define VRTs as training devices which attempt to accommodate the muscle changing level of force output throughout the ROM by changing the provided external resistance. Among modalities of VRT, CAM-Nautilus Machine (NM) and, recently, Elastic Resistance exercises (ER) have increasingly gained popularity among athletes and recreational lifters (Anderson et al., 2008; Page et al., 1993; Treiber et al., 1998). However, to date no research has addressed the pattern and rate of applied load and muscle activation during intensive ER and NM exercises.

Elastic resistance is well established as an affordable and effective mode of training in rehabilitation and fitness settings (Page and Ellenbecker, 2003; Hintermeister et al., 1998; Schulthies et al., 1998). However, there is controversial evidence regarding utilization of ER for high intensity training protocols (Treiber et al., 1998). Reportedly, ER cannot lead muscle to its maximal activation level due to inadequate external force (Page et al., 1993; Matheson et al., 2001). Elastic tubing is being produced in several colour-codes and each colour denotes a specific resistance (Patterson et al., 2001; Simoneau et al., 2001). Hughes (1999) reported the magnitude of resistance in elastic device (Hygienic Corporation, Akron, Ohio) from 3.3N to 70.1N for yellow and silver colour when elastic materials were at 18 and 159% of deformation from resting length, respectively. Based on this viewpoint, utilizing ER has been confined to the initial and intermediate stages of rehabilitation protocols (Hintermeister et al., 1998; Hopkins et al., 1999).

Accordingly, in this investigation two strategies are employed to increase the magnitude of elastic force. Firstly, additional elastic bands are utilized in parallel to the current unit (Page et al., 1993). Secondly, the initial length of the elastic material are reduced to enhance the magnitude of force at the beginning of concentric phase (Treiber et al., 1998; Hodges, 2006). We hypothesize that applying these strategies can improve the tensile force in ER and result in achieving comparable muscle activation as NM during performing high intensity knee extension exercise. The results of this investigation may facilitate better understanding for prescribing high intensity training protocols in contribution of these two modes of exercise. The purpose of this investigation, therefore, was to quantify and compare the magnitude of applied load and muscle activation (EMG) during 8-RM seated knee extension in contribution of ER and NM.

## Methods

### Subjects

Seven female (mean ± SD; 22.4 ± 4.7 age, 60.05 ± 6.17 kg, 158 ± 3 cm) and 9 male (24.0 ± 3.6 age, 78.14 ± 7.2 kg, 174 ± 7cm) healthy volunteers were recruited for this study. None of the subjects had experience of participating in any resistance training program in the past 12 months. This study was approved by the ethics committee of Sports Centre, University of Malaya and all participants signed the informed consent forms.

### Experimental design

The measurement scheme of the present investigation was started with an orientation session in which the benefits and risks of participating in the study were clarified for the participants. After seven days, subjects attended the main testing session. To avoid inaccurate positioning of electrodes from day to day testing, all data were collected within one experimental session. The order of the measurement was randomized across 3 exercise modalities. The three types of resistance exercise in this research consisted of NM, elastic tubing with initial length (E0) and elastic tubing with 30% decrement of initial length (E30). The subjects undertook 8-RM knee extension trials by 3 modes of training with 5 min recovery periods between training modalities. Ten subjects were randomly selected to duplicate similar procedure after 5 days to compute the test-retest reliability on two occasions.

### Instrumentation

EMG muscle activation was measured with a sample rate of 1000 Hz using a 16-bit acquisition mode with an eight-channel TeleMyo™ 2400T G2 EMG system (Noraxon, Scottsdale, Arizona, USA). Pre-gelled silver/silver chloride adhesive surface electrodes (Meditrace, Canada) were used to detect electromyographic signals. EMG signals were passed through a build-in preamplifier leads (Input impedance of 500 MΩ common mode rejection ratio of 130 dB). Receiver unit collected the telemetry signals from the receiver amplified and filtered (15 Hz to 1000 Hz) the signals. Range of motion of dominant knee was monitored using a 2-D electrogoniometer (Noraxon, Scottsdale, Arizona, USA). In order to avoid any biomechanical interference in position of the subjects, the nautilus knee extension chair (Nautilus, Vancouver, WA) was used for ER exercise testing as well. The lever arm in nautilus machine and the ankle cuff in elastic device were equipped with a force transducer (Noraxon, Scottsdale, Arizona, USA) to measure magnitude of applied force. Data were collected and synchronized using the data acquisition package Myoresearch-XP, Master Edition (Noraxon, Scottsdale, Arizona, USA).

### Measurement procedure

A week prior to the experiment all subjects attended an orientation session in which, for familiarization, they were required to practice Maximal Voluntary Isometric Contractions (MVIC) and 8-RM trials with three modes of exercise. The external load was either added or removed to achieve the actual 8-RM for each mode of training (Treiber et al., 1998). This goal for elastic material was fulfilled by examining different combination of elastic colour codes (but similar length) to meet the actual number of repetitions (8-RM). The initial length of elastic material (Hygienic Corporation, Akron, OH) was determined for every subject by measuring the distance from the origin of the elastic device (elastic tubing was anchored to the base of the NM chair) to the axis (ankle cuff). In this way, the combination of colour code as well as the length of elastic device was personalized for each subject. In addition, subjects were asked not to participate in any training 48 hours before the main testing session.

The main testing session for each participant started at 8 a.m. The warm up was performed comprising static stretching and 5 min cycling on an ergometer with a self-selected pace. Following the warm up, the subjects were allowed to rest for 5 min, during which time the electrodes were positioned parallel to the direction of the Vastus Lateralis (VL) muscle fibres above the midpoint of the muscle belly as assessed by palpation. The ground electrode was placed on the patella bone. Before placement of electrodes, the subject’s skin was shaved and cleaned with alcohol to reduce skin impedance.

Each subject was then seated on the knee extension NM according to the procedure reported by Manning et al. (1990). A five-second baseline signal was collected from the muscles to ensure no artefacts existed. All the subjects completed 3 trials of unilateral MVIC with the dominant leg. For this aim, the NM chair was set to obtain a 120° knee extension. Each trial lasted 5 seconds and two minutes rest intervals were assigned between trials to prevent fatigue (Matheson et al., 2001). The MVIC was determined as an average of amplitude over one second window of the highest rectified EMG signals (automatically selected by Myoresearch-XP). It was used as a reference value for normalizing muscle activation data during dynamic exercises (%MVIC).

The subjects then completed 8-RM knee extension trials by 3 modes of training with 5 min recovery periods between training modalities. Repetitions were completed within 80 to 180º of knee extension with the cadence of 1.5 s concentric and 1.5 s eccentric, set by a metronome. One second pause between every repetition was assigned to avoid potential stretch-shortening cycle interference in the concentric-eccentric merging phase. To control the position of shank during dynamic contractions, two laser beams connected to an alarm system limited the range of motion at each extremity. Therefore, an alarm sounded if subject’s foot would touch laser spectrums. An attempt at 8-RM was deemed successful if all repetitions were performed in accordance with the pace of the metronome without any compromise in ROM. Therefore, the cadence of performing exercises and the range of motion were limiting factors on the amount of external resistance. Ten subjects were randomly selected to duplicate a similar procedure after 5 days to compute reliability of testing over two occasions. The test-retest reliability for magnitude of external force at each phase of contraction was 0.92, 0.89 and 0.95 for E30, E0 and NM, respectively.

Although data were collected from all repetitions during 8-RM, the first (initial), the 5^th^ (middle) and the 8^th^ (last) repetitions were selected for further analysis. Appointed repetitions were partitioned into concentric and eccentric components based on the end points determined by the electrogoniometer traces. Then, the value of every component was divided into 3 equal phases. Accordingly, the division of movement into six phases (3 concentric and 3 eccentric) across the entire ROM (80 – 180° of knee extension) comprised of 80 – 113°, 113 – 146° and 146 – 180° for the 1^st^, 2^nd^ and 3^rd^ concentric phases respectively, and in a reverse order for the 4^th^, 5^th^ and 6^th^ phases of eccentric contraction. The Root Mean Square (RMS) of rectified EMG signals and the average of external force (N) were calculated for each phase. The average of 6 phases for the EMG and the force were then used to represent the value of each repetition. Then, the values of 1^st^, 5^th^ and 8^th^ repetitions were used for calculating the total value for each exercise modality (6 phases × 3 repetitions).

### Statistical Analysis

Differences in EMG and external force values were examined within various phases (1 to 6), repetitions (1, 5 and 8) and modalities of exercise (NM, E0 and E30) using a 6 × 3 × 3 Repeated Measure Analysis of Variance (ANOVA). If significant results were obtained from ANOVA, a series of pair sample *t-tests* were used to compare analogous phases and repetitions among modalities of exercise. Significance was defined as *p < .05*.

## Results

### Applied Force

**Figure 1:**
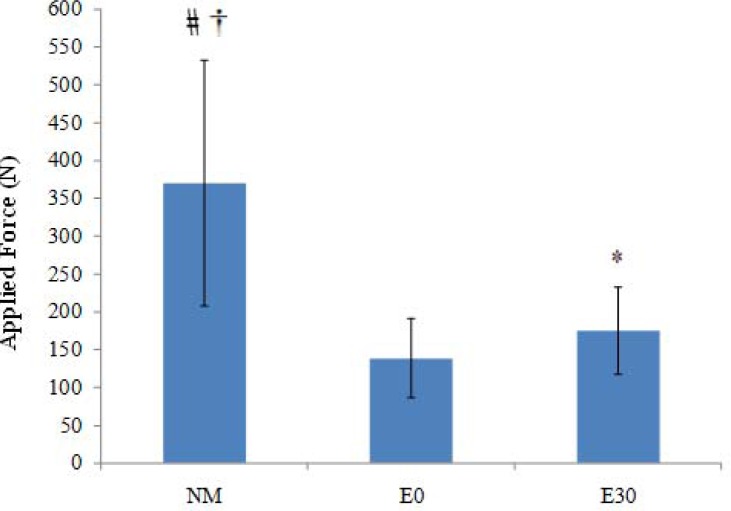
Total average Applied Force ± SD (N) within various exercise modes. The value of every column includes the average of 3 repetitions and 6 phases.* = E30 is significantly higher than E0. † = NM is significantly higher than E0. # = NM is significantly higher than E30.

The data addressing the magnitude of applied force for the main effects (phases, repetitions and exercise types) are listed in Table 1. The analysis of variance demonstrated statistically significant values for the interaction of the main effect phases × repetitions × training modes (*p < .01*). Subsequently, a series of pair sample *t-tests* among Total Average force (the value of each exercise mode comprised of 3 rep × 6 phases) indicated that there was a considerable difference between NM and both ER modalities (Figure 1). In addition, E30 exhibited a significantly higher overall value compared with E0 (*p < .05*).

**Figure 2:**
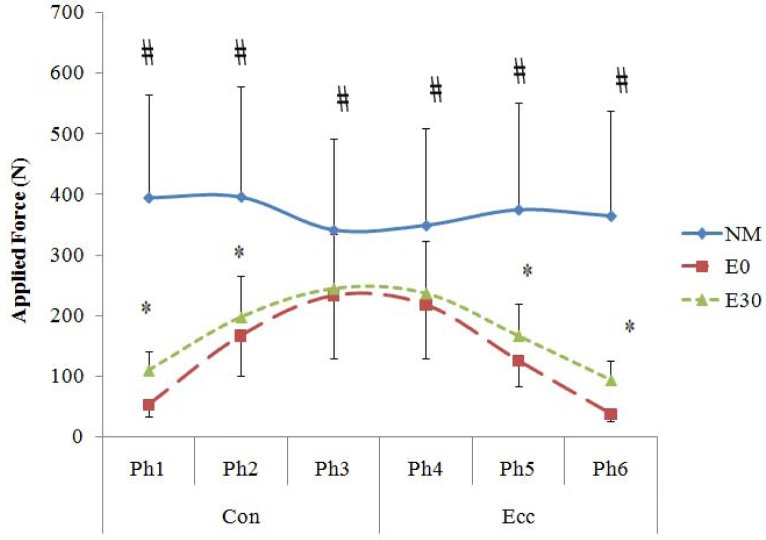
*Mean ± SD for Applied Force (N) within various phases of exercise modes. The value of every phase includes the average of 1^st^+ 5^th^+ 8^th^repetitions. # = NM is significantly higher than E0 and E30.* * = E30 is significantly higher than E0.

The results concerning the pattern of applied force during each mode of exercises are depicted in Figure 2. The force-angle relationship was an inverted “U” for E30 and E0 throughout the whole ROM. The data indicate that during the concentric contraction both types of ER devices provided significantly greater external force in the 2^nd^ and 3^rd^ phases compared with the 1^st^ phase; though, no significant difference was observed between the 2^nd^ and 3^rd^ phases. During eccentric contraction however a significant decline in magnitude of external force was observed during which the 6^th^ phase < the 5^th^ phase < 4^th^ phase. However, for NM the only systematic change was an insignificant decline in magnitude of force toward the 3^rd^ and 4^th^ phases (*p* < *.05*).

**Figure 3:**
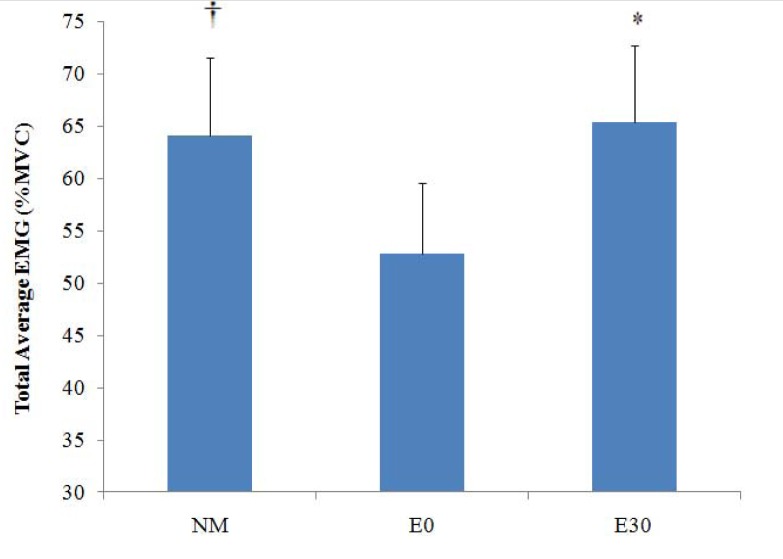
Total average EMG ± SD (%MVC) within various exercise modes. The value of every column includes the average of 3 repetitions and 6 phases.* = E30 is significantly higher than E0.***†****= NM is significantly higher than E0.*

***EMG.*** The results addressing the means and standard deviations for the main effects (phases, repetitions and exercise types) are listed in a hierarchical structure in Table 2. Analysis of variance demonstrated a significant value for the interaction effects of phases × repetitions × training mode (*p = .025; p < .05*). Subsequently, a series of pair sample *t-tests* among Total Average EMG (the value of each exercise mode comprised of 3 rep × 6 phases) indicated that there was no significant difference between E30 and NM (Figure 3); though, either of these exercise types exhibited a significantly higher value compared with E0 (all *p < .05*).

**Figure 4:**
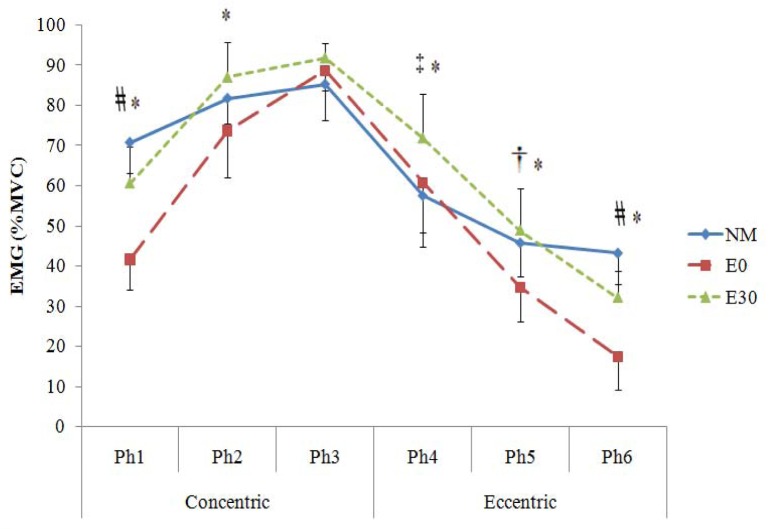
*Mean ± SD for EMG (%MVC) within various phases of exercise modes. The value of every phase includes the average of 1^st^+ 5^th^+ 8^th^repetitions****. †****= NM is significantly higher than E0.****‡***= E30 is significantly higher than NM. # = NM is significantly higher than E0 and E30. * = E30 is significantly higher than E0.

The EMG values for various phases of contraction in the three exercise types are presented graphically in Figure 4. These results indicated that in the 1^st^ and 6^th^ phases NM generated significantly higher muscle activation than both elastic modalities and E30 attained a significantly higher value than E0 (*p* < *.05*). In the 2^nd^ through 5^th^ phases only E30 showed a significantly higher muscle activity than E0, but no other significant differences were observed (*p* < *.05*).

## Discussion

The purpose of this descriptive study was to quantify and compare the EMG activity and the applied load during high intensity seated leg extension exercises by NM and ER devices. To enhance the provided external force by ER device, two strategies were employed: firstly, additional elastic bands were utilized in parallel to the current unit to develop overall tensile force (Page et al., 1993); secondly, the initial length of the elastic material was reduced to enhance the magnitude of force at the beginning of the concentric phase (Treiber et al., 1998; Hodges, 2006). The data presented in Figure 3 suggested that applying a combination of these two strategies could result in producing equal overall muscle activation (EMG) by E30 compared with NM. Furthermore, reducing the initial length of the elastic device (the only source of difference between E30 and E0) could significantly improve muscle activation (28.8%) and applied force (25.8%) for E30 compared with E0.

The effectiveness of these strategies becomes even more evident when the pattern of muscle activity is compared across the three types of training. As depicted in Figure 4, significantly higher EMG was achieved by E30 compared with E0 in the 1^st^, 2^nd^, 4^th^, 5^th^ and 6^th^ segments. In addition, E30 generated muscle activation equal to that of NM in the 2^nd^ to 5^th^ segments. Such results suggest E30 as a modified form of elastic device which can partially overcome a chronic drawback of ER exercise in eliciting adequate muscle activation throughout the ROM, particularly at the beginning of the concentric phase (Lim and Chow, 1998; Matheson et al., 2001).

Despite the above findings, significantly less EMG activity at the 1^st^ and 6^th^ phases and considerably less applied force of E30 compared with NM across the entire ROM indicate that caution should be taken before accepting E30 as an inclusive mode of training for high intensity resistance exercise protocols. In fact, despite reducing by 30% the initial length, E30 could not provide adequate external resistance to meet the force generating capability of quadriceps muscle at these two particular segments. This finding is in accordance with the results reported by Hodges (2006). He demonstrated that even shortened elastic tubing provides less average resistance and consequently lower neuromuscular adaptation than that of traditional free weights at the beginning of concentric and end of eccentric segments of motion. These results point to the need for more studies to elucidate if a reduction of initial elastic device length (e.g. 40% or 50%) would result in more muscle activity and provide elastic force in these segments of motion. A unique aspect of the data in the present study is the observation of equal Total Average EMG activity between E30 and NM, despite a considerably smaller external load (111.4%) that was employed during E30 compared with NM (Figure 1). This result propounds the question “how could a smaller external force in E30 elicit a similar rate of muscle activation compared with NM?” This discrepancy was also evident across a whole range of motion (Figures 2 and 4) where a higher applied force within NM exercise was not reflected in EMG values. Interestingly, the data indicated that although extensively greater external load was employed by NM during the whole ROM, insignificantly higher EMG activity was detected for E30 compared with NM in the 2^nd^, 3^rd^ and 5^th^ phases. In the 4^th^ phase E30 demonstrated significantly higher muscle activity than NM. On the other hand, E0 demonstrated an equal EMG activity to NM in the 2^nd^, 3^rd^ and 4^th^ phases of contraction, although 137.1%, 46.7% and 60% greater load was employed by NM than E0 during these phases, respectively.

The reason behind this relatively higher EMG for E30 is unclear. However, since distal extremity of lower leg (ankle) had a higher degree of freedom during knee extension by ER device (compared with restricted-unidirectional NM lever arm) more control over the movement was required to keep lower leg motion aligned in sagittal plane (McCaw & Friday, 1994). Therefore, muscle activity during E30 could have been partially devoted to control lower leg movements throughout the assigned range of motion. In advocate of this idea Richards and Dawson (2009) indicated that performing exercises in a multiaxial direction could potentially change the rate of muscle activation via altering motor unit recruitment. Overall, the data supported the idea of Bosco et al. (2000) regarding the concept of exercise intensity. They stated that contrary to the classical thought which had defined exercise intensity as the magnitude of the load employed, it must have been defined as the rate of the work performed.

In the 1^st^ and 6^th^ phases, E30 and E0 generated significantly less EMG activity compared with NM (Figure 4). This result could be attributed to the necessity of less muscle effort to overcome the inertia of much lower external load in ER exercises during the early concentric and late eccentric phases of contraction. Nonetheless, the findings of the present study highlighted the effect of reducing the initial length of elastic material in achieving significantly higher muscle activation and applied lead by elastic resistance device (Figures 2 and 4). The data demonstrated dramatically higher EMG values for E30 compared with E0 in all phases of contraction, except in the 3^rd^ phase in which equal EMG readings was observed between the two modes of training. Based on similar finding, Hodges (2006) concluded that after reducing the initial length of elastic material, a shifting occurs in the distribution of muscle tension from late concentric to early concentric and from early eccentric to late eccentric range of motion. Accordingly, E30 exhibited significantly higher EMG than E0 in the 1^st^ (48%) and the 6^th^ (84.31%) phases. These data disclose the importance of reducing the initial length as an essential strategy to develop muscle activation by ER devices.

## Conclusion

Many athletes rather use various modalities of resistance exercise (e.g. free weights, pulley machines, isokinetic dynamometers, elastic resistance, etc) within their conditioning program with the prevailing view that each type of strength training offers a unique mechanical and physiological muscle stimulation (Welsch et al., 2005). On this basis, undertaking several types of resistance exercise might facilitate better development of the muscle performance. Based on equal average EMG between E30 and NM, the findings of the present study suggest that E30 could be an alternative to the use of NM in high exercise intensity (8-RM). However, since NM displayed higher EMG compared with E30 in the early concentric and late eccentric phases and E30 demonstrated higher muscle activation in the late concentric and early eccentric phases of contraction, a training protocol comprised of both modes of exercise seems to be ideal.

## Figures and Tables

**Table 1:** Magnitude of applied force within various phases of motion for three types of exercise

**Force (N)**									
NM				E0			E30		
Total Average of 3 Rep × 6 Phases				Total Average of 3 Rep × 6 Phases			Total Average of 3 Rep × 6 phases		
370.3* (162.5)				138.8 (52.3)			175.0‡ (57.7)		
Average of all 6 phases				Average of all 6 phases			Average of all 6 phases		
rep 1		rep 5	rep 8	rep 1	rep 5	rep 8	rep 1	rep 5	rep 8
361.5*† (152.1)		374.6* † (165.5)	374.7*† (170.6)	134.0 (51.1)	140.0 (52.5)	141.9 (54.2)	174.0‡ (57.3)	177.3‡ (56.9)	173.7‡ (60.6)
Mean (±SD) of Applied Force values of every segments (N=16)									
1	328.5*† (145.9)	412.6*† (173.5)	443.2*† (223.7)	46.4 (22.0)	56.0 (21.8)	55.5 (21.8)	116.1‡ (43.4)	104.7‡ (33.1)	107.7‡ (28.3)
2	389.7*† (179.9)	395.5*† (184.0)	403.1*† (185.1)	154.8 (66.7)	167.2 (60.0)	178.3 (73.3)	193.3‡ (66.4)	195.4‡ (76.2)	204.7‡ (66.9)
3	354.1*† (151.8)	347.5*† (161.5)	323.4*† (135.7)	227.7 (98.9)	233.7 (103.3)	236.3 (106.1)	245.2 (89.4)	240.8 (92.3)	248.9 (90.7)
4	352.6*† (163.9)	348.4*† (159.2)	346.9*† (157.8)	217.1 (87.8)	218.9 (94.1)	217.3 (85.5)	233.0 (82.2)	240.5 (86.7)	238.5 (88.1)
5	369.5*† (172.7)	376.5*† (173.8)	379.0*† (180.8)	123.0 (44.1)	124.1 (43.9)	129.3 (45.0)	160.4‡ (47.2)	178.8‡ (59.9)	161.5‡ (63.3)
6	374.4*† (174.2)	367.2*† (175.7)	352.6*† (170.3)	39.1 (15.4)	40.0 (15.0)	34.5 (14.5)	95.9‡ (28.5)	103.7‡ (37.9)	81.1‡ (47.9)

NOTE. Mean (±SD) of applied force for various phases of motion (1–6), Average of 6 phases for every repetition (rep) and To Average of 3 Repetitions for every mode of exercises.

* = NM is significantly higher than E0.

‡ = E30 is significantly higher than E0.

† =NM is significantly higher than E30 (all p < 0.05).

## References

[b1-jhk-30-5] American College of Sports Medicine (2002). Position stand: Progression models in resistance training for healthy adults. Medicine and Science in Sports and Exercise.

[b2-jhk-30-5] Anderson CE, Sforzo GA, Sigg JA (2008). The Effects of Combining Elastic and Free Weight Resistance on Strength and Power in Athletes. The Journal of Strength & Conditioning Research.

[b3-jhk-30-5] Bosco C, Colli R, Bonomi R, Von Duvillard SP, Viru A (2000). Monitoring strength training: neuromuscular and hormonal profile. Medicine & Science in Sports & Exercise.

[b4-jhk-30-5] Elliott BC, Wilson GJ, Kerr GK (1989). A biomechanical analysis of the sticking region in the bench press. Medicine & Science in Sports & Exercise.

[b5-jhk-30-5] Fleck SJ, Kraemer WJ (2004). Designing Resistance Training Program.

[b6-jhk-30-5] Hintermeister RA, Bey MJ, Lange GW, Steadman JR, Dillman CJ (1998). Quantification of Elastic Resistance Knee Rehabilitation Exercises. journal of Orthopedic and Sport Physical Therapy.

[b7-jhk-30-5] Hodges GN (2006). The effect of movement strategy and elastic starting strain on shoulder resultant joint moment during elastic resistance exercise.

[b8-jhk-30-5] Hopkins JT, Christopher DI, Michelle AS, Susan DB (1999). An Electromyographic Comparison of 4 Closed Chain Exercises. jorunal of athletic training.

[b9-jhk-30-5] Hughes CJ, Hurd K, Jones A, Sprigle S (1999). Resistance Properties of Thera-Band® Tubing During Shoulder Abduction Exercise. Journal of Orthopedic and Sports Physical Therapy.

[b10-jhk-30-5] Lim Y, Chow J (1998). Electromyographic comparison of biceps curls performance using a dumbbell and an elastic tubing. The North American congress on biomechanics.

[b11-jhk-30-5] Manning RJ, Graves JE, Carpenter DM, Leggett SH, Pollock ML (1990). Constant vs variable resistance knee extension training. Medicine & Science in Sports & Exercise.

[b12-jhk-30-5] Matheson JW, Kernozek TW, Fater DCW, Davies GJ (2001). Electromyographic activity and applied load during seated quadriceps exercises. Medicine & Science in Sports & Exercise.

[b13-jhk-30-5] McCaw ST, Friday JJ (1994). A Comparison of Muscle Activity Between a Free Weight and Machine Bench Press. The Journal of Strength & Conditioning Research.

[b14-jhk-30-5] Page JL, Ben A, Robert B, Robert C, Robert C (1993). Posterior Rotator Cuff Strengthening Using Theraband® in a Functional Diagonal Pattern in Collegiate Baseball Pitchers. Journal of Athletic Training.

[b15-jhk-30-5] Page P, Ellenbecker T (2003). The Scientific and Clinical Application of Elastic Resistance.

[b16-jhk-30-5] Patterson RM, Stegink Jansen CW, Hogan HA, Nassif MD (2001). Material Properties of Thera-Band Tubing. Physical Therapy.

[b17-jhk-30-5] Richards JA, Dawson TA (2009). Optimizing Exercise Outcomes: The Efficacy of Resistance Training Using Conventional vs. Novel Movement Arcs. Journal of Strength and Conditioning Research.

[b18-jhk-30-5] Schulthies SS, Ricard MD, Alexander KJ, Myrer JW (1998). An Electromyographic Investigation of 4 Elastic-Tubing Closed Kinetic Chain Exercises after Anterior Cruciate Ligament Reconstruction. Journal of Athletic Trainining.

[b19-jhk-30-5] Simoneau GG, Bereda Shellie M, Sobush Dennis C, Starsky Andrew J (2001). Biomechanics of Elastic Resistance in Therapeutic Exercise Programs. Journal of Orthopedic and Sports Physical Therapy.

[b20-jhk-30-5] Treiber FA, Lott J, Duncan J, Slavens G, Davis H (1998). Effects of Theraband and Lightweight Dumbbell Training on Shoulder Rotation Torque and Serve Performance in College Tennis Players. The American Journal of Sports Medicine.

[b21-jhk-30-5] Wallace BJ, Winchester JB, McGuigan MR (2006). Effects of Elastic Bands on Force and Power Characteristics During the Back Squat Exercise. The Journal of Strength & Conditioning Research.

[b22-jhk-30-5] Welsch EA, Bird M, Mayhew JL (2005). Electromyographic Activity of the Pectoralis Major and Anterior Deltoid Muscles During Three Upper-Body Lifts. The Journal of Strength & Conditioning Research.

